# The time course of spontaneous closure of idiopathic full-thickness macular holes

**DOI:** 10.1007/s00417-024-06465-2

**Published:** 2024-04-08

**Authors:** Jonas Neubauer, Faik Gelisken, Taylan Ozturk, Karl-Ulrich Bartz-Schmidt, Spyridon Dimopoulos

**Affiliations:** 1 Department of Ophthalmology, Eberhard Karls University, Elfriede-Aulhorn-Straße 7, 72076 Tuebingen, Germany; 2 Department of Ophthalmology, Dokuz Eylul University, Izmir, Turkey

**Keywords:** Macular Hole, Full Thickness Macular Hole, Spontaneous Closure, Time Course, Re-opening, Watch and Wait

## Abstract

**Purpose:**

Spontaneous closure of idiopathic full-thickness macular holes (iFTMH) has been reported regularly. However, little is known about its probability and timeline.

**Methods:**

In this retrospective study all consecutive patients who presented between August 2008 and August 2019 were screened for the presence of a macular hole and only iFTMHs were included. The primary outcome measure was the spontaneous closure of the iFTMH.

**Results:**

Of 1256 eyes with macular holes, 338 fulfilled the inclusion criteria. Spontaneous closure of the iFTMH was detected in 31 eyes (9.2%) with a median time of 44 days after diagnosis. Eyes exhibiting spontaneous closure demonstrated a higher baseline best-corrected visual-acuity (BCVA) and smaller iFTMH diameter (*p* < 0.0001 and *p* < 0.0001, respectively). The mean BCVA improved from 0.4 logMAR (SD ± 0.21) to 0.29 logMAR (SD ± 0.20) after spontaneous closure (*p* = 0.031). The iFTMH diameter was positively correlated with the time to spontaneous closure (Pearson-r = 0.37, *p* = 0.0377). Spontaneously closed iFTMHs reopened in 16% (*n* = 5) of cases, with a median of 136 days after closure. A logistic regression model showed the hole diameter was associated with spontaneous closure (odds-Ratio 0.97, 95%CI [0.96, 0.98]). The Kaplan–Meier-Curve revealed that approximately 25% of small-iFTMH (*n* = 124) and 55% of iFTMH with a diameter < 150µm (*n* = 48) closed spontaneously within two months.

**Conclusion:**

The established gold-standard for the treatment of iFTMHs is macular surgery. However, the potential for spontaneous closure of small iFTMHs must be acknowledged. Therefore, if surgical treatment is delayed in individual cases, close observation is recommended.


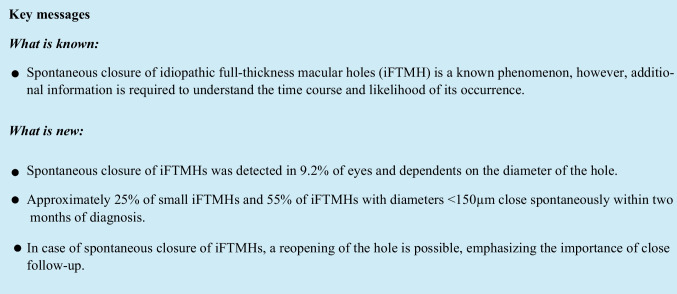


## Introduction

An idiopathic full-thickness macular hole (iFTMH) is a vitreomacular interface disease with an opening of all neurosensory retinal layers in the fovea, leading to vision loss, metamorphopsia and central scotoma. It is more frequently observed in women over the age of 65, with an incidence of about eight eyes per 100,000 per annum [1]. Following the report of the first surgical closure of iFTMHs by Kelly and Wendell, advanced vitreoretinal surgical techniques contributed to successful postoperative restoration of the foveal microstructure with improvement of the vision [2–7]. There have been also efforts to achieve closure of iFTMH by intravitreal pharmacotherapy, which resulted in a favorable outcome in selected cases [8]. Currently, the first-line therapy for the iFTMH is macular surgery.

Although the macular surgery is a safe and effective, spontaneous closure of iFTMHs has been reported repeatedly over the years [9–13]. Before the introduction of Optical Coherence Tomography (OCT), spontaneous closure of iFTMHs was described in several studies, ranging from 0% to 15.8% [14–16]. However, given the difficulty of diagnosing small iFTMHs by funduscopy, these studies are subject to possible bias. To our knowledge, few studies have investigated the spontaneous closure of iFTMH using OCT imaging [17–19].

While the aforementioned reports focused on the rates of spontaneous closure, the dynamics of this phenomenon are also critical, as delayed surgery is associated with poor prognosis [20, 21]. Therefore, the aim of this study was to investigate the factors related to the spontaneous closure and the time course of this phenomenon in particular.

## Methods

We retrospectively reviewed the electronical medical charts of all patients presenting between August 2008 and August 2019 at the Department of Ophthalmology of the University of Tuebingen and included 1256 eyes diagnosed with an iFTMH. All patients underwent complete ophthalmologic examination, including best-corrected visual acuity (BCVA), slit-lamp biomicroscopy and dilated funduscopic evaluation. Spectral domain OCT (Heidelberg Engineering GmbH, Heidelberg, Germany) was performed at the first presentation and in all follow-up examinations. Patients received a volume scan (25 cross sections with a distance of 61 µm) and six or 12 radial scans. The primary outcome measure was a spontaneous closure of the iFTMH detected on OCT imaging. Inclusion criteria were: 1- Available SD-OCT at the first presentation and follow-up examinations; 2- SD-OCT examination one day before the scheduled macular surgery. Exclusion criteria were: 1- Previous intravitreal pharmacotherapy; 2- Ocular diseases that could interfere with morphology of the macula or the visual prognosis; 3- Ocular trauma; 4- Previous vitreoretinal surgery, laser photocoagulation or cryosurgery; 5- Myopia of less than minus six diopters (< -6 Diopters); 6- Missed OCT-examination or poor image quality; 7- Secondary full-thickness macular holes. The diagnosis of an iFTMH was based on the OCT imaging and the classification by the International Vitreomacular Traction Study (IVTS) Group was used for grading [22]. The iFTMH diameter was determined by measuring the smallest distance at the mid-retina on the selected OCT scan that captured the widest cross-sectional image of the macular hole [22]. To ensure the reliability of the measurements, 30 eyes were randomly selected, and the diameter of the iFTMH was measured by a second ophthalmologist without revealing the value of the first measurement, resulting in an excellent intraclass correlation of 0.95. The presence of vitreomacular traction and epiretinal membrane was documented. Patients were informed about the natural course and therapy options, including close observation in case of a small iFTMH and good baseline BCVA, intravitreal pharmacotherapy in selected cases, and vitrectomy with macular surgery. The surgery was scheduled as soon as possible, and the OCT examination was repeated on the day before the surgery. For the creation of Kaplan–Meier curves all available data were considered. In the absence of spontaneous closure, the last OCT scan with an iFTMH was noted as the date of censoring.

The iFTMHs were graded as proposed by the International Vitreomacular Traction Study Group (IVTS) in small (≤ 250 µm), medium (251-400 µm) and large (> 400 µm) iFTMHs and depending on the presence/absence of vitreofoveal traction [22]. Visual acuity was analyzed using logMAR. Statistical analysis was performed using R and Prism 6, GraphPad. The D'Agostino and Pearson omnibus normality test, Fisher’s test, Chi-square test with Yates’ correction and Mann–Whitney U test were used and statistical significance was assumed for *p* < 0.05. This study was performed according to the principles of the Declaration of Helsinki and it was approved by the Ethics Committee of the University of Tuebingen (Project Number 215/2020BO2).

## Results

Of the total 1256 eyes with iFTMH, 338 fulfilled the inclusion criteria. A total of 918 eyes were excluded (549 eyes with previous intraocular surgery or associated macular diseases, 360 eyes without OCT examination and nine eyes with low-quality of the OCTs). The study cohort comprised 338 eyes of 324 patients (199 women) with a mean age of 68.9 years (range 40 – 90 years). Bilateral involvement was observed in 14 patients (4.1%) at the first visit or during the follow-up period. The time interval between the diagnosis and surgery was in median 34 days (range 4–618). Of the patients without spontaneous closure (*n* = 307), 279 (90.9%) agreed to have surgery. A total of 28 patients (28 eyes, 9.1%) refused surgery. The median follow-up time of these patients was 149 days (range 16–1849). In the spontaneous closure group, two out of 31 patients refused surgery. No significant difference was found between the two groups regarding surgery refusal (*p* = 1). Table 1 summarizes the baseline characteristics of the study population.

**Table 1:** Baseline characteristics of the study population

	No Spontaneous Closure (*n* = 307)	Spontaneous Closure (*n* = 31)	
Age, mean	69,11 (SD 7,98) (range, 40–89 years)	66,77 (SD 9,24) (range, 54–90 years)	*p* = 0.13^1^
Sex	184 female (60%)	15 female (48%)	*p* = 0.25^2^
	123 male (40%)	16 male (52%)	
Lens			
Phakic	223 (73%)	23 (74%)	*p* = 1^2^
Pseudophakic	84 (27%)	8 (26%)	
BCVA Baseline (mean)	0.68 (SD 0.27)	0.40 (SD 0.21)	*p* < 0.0001^3^
Gass Classification			
2	94 (31%)	31 (100%)	
3	70 (23%)	-	
4	142 (46%)	-	
IVTS Classification*			
Small without VMT	62 (20%)	17 (55%)	
Small with VMT	31 (10%)	14 (45%)	
Medium without VMT	75 (24%)	-	
Medium with VMT	39 (13%)	-	
Large without VMT	78 (25%)	-	
Large with VMT	22 (7%)	-	
Vitreomacular Traction			
No	215 (70%)	17 (55%)	*p* = 0.10^2^
Yes	92 (30%)	14 (45%)	
Epiretinal Membrane			
No	224 (73%)	20 (65%)	*p* = 0.40^2^
Yes	83 (27%)	11 (35%)	
Mean Diameter (µm)	345,6 (SD 161,6)	98,9 (SD 58,32)	*p* < 0,0001^3^
Median Time to Spontaneous Closure (days)		44 (range, 5–1013)	

BCVA: Best-corrected visual acuity

VMT: Vitreo-macular traction

SD: Standard deviation

^1^ Unpaired t-test (with D'Agostino & Pearson Omnibus normality test)

^2^ Fisher's exact test

^3^ Wilcoxon-Mann–Whitney-Test

^*^ IVTS Classification: Small ≤ 250 μm, Medium > 250 μm and ≤ 400 μm, Large > 400 μm

**Fig. 1:**
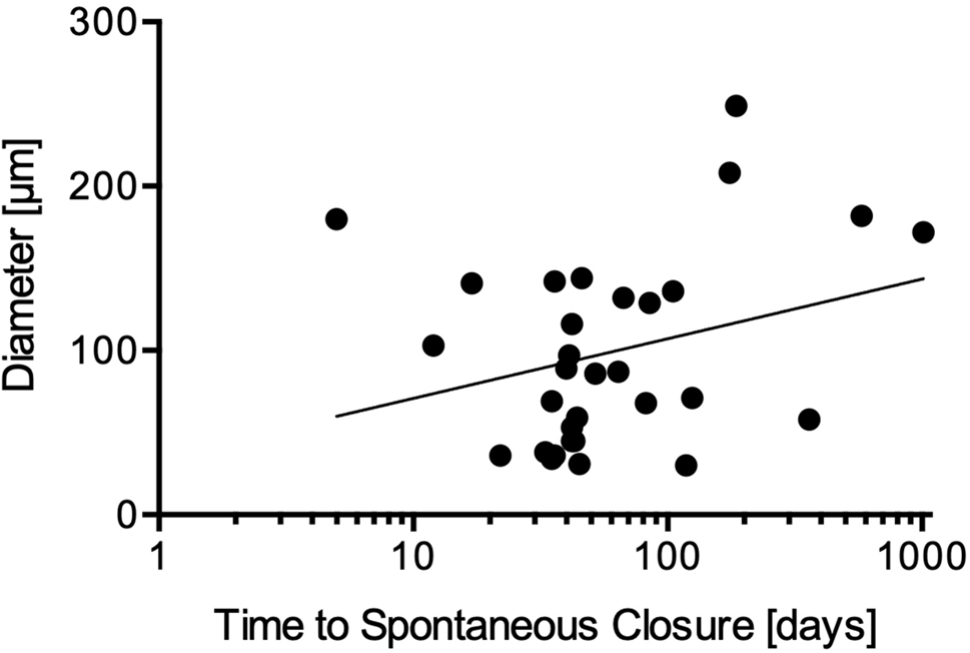
Semi-logarithmic regression of idiopathic full-thickness macular hole (iFTMH) diameter and the time to spontaneous closure (*n* = 31). A smaller iFTMH diameter was significantly correlated with a shorter time to spontaneous closure (Pearson r = 0.37, *p* = 0.0377)

Spontaneous closure of the iFTMH was detected in 31 eyes (9.2%) of 30 patients with a median time of 44 days (range 5–1013) after diagnosing the iFTMH. One patient showed spontaneous closure in both eyes. No significant differences were observed in terms of diameter or time to spontaneous closure between patients with and without vitreomacular traction (*p* = 0.40 and *p* = 0.55, respectively). Eyes with spontaneous closure had a higher baseline BCVA and smaller iFTMH diameter (*p* < 0.0001 and *p* < 0.0001, respectively), and there was no statistically significant difference in age, gender, lens status, frequency of vitreofoveal traction or the presence of an epiretinal membrane (*p* = 0.13, *p* = 0.25, *p* = 1, *p* = 0.10 and *p* = 0.40, respectively). The mean BCVA improved significantly from 0.4 logMAR (SD ± 0.21) to 0.29 logMAR (SD ± 0.20) after spontaneous closure (*p* = 0.031). In eyes with spontaneous closure, the diameter of the iFTMH was positively correlated with the time to spontaneous closure (Pearson r = 0.37, *p* = 0.0377) (Fig. 1).

In the logistic regression analysis of age, baseline BCVA, lens status, status of vitreofoveal traction and iFTMH diameter, only the diameter [µm] was found to be significantly associated with spontaneous closure (odds Ratio 0.97, 95%CI [0.96, 0.98]). The mean diameter of the spontaneously closed iFTHM was 98.9 µm (range 30-249 µm), corresponding to a small iFTMH. The median follow-up after spontaneous closure (available for 23 patients, 74.1%) was 406 days (range 21 to 4161 days). A re-opening of the spontaneous closed iFTMH was found in five eyes (16.1%) after a median of 136 days after closure (range 23 to 708 days).

**Fig. 2:**
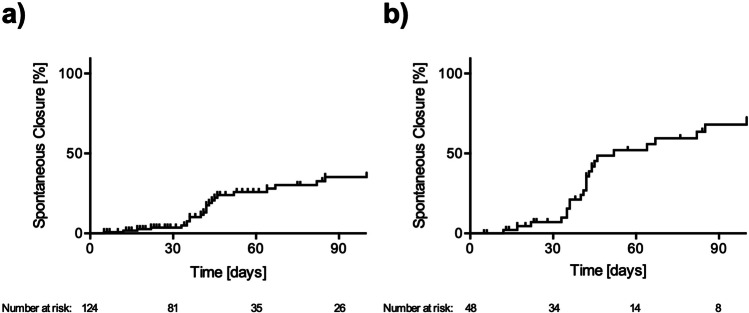
Kaplan–Meier-Plot of spontaneous closure. Upward blips represent censored patients. **a** Approximately 25% of idiopathic full-thickness macular holes (iFTMH) with a diameter ≤ 250 µm (*n* = 124) close spontaneously within two months of diagnosis. **b** 55% of iFTMHs with a diameter ≤ 149 µm (*n* = 48) close spontaneously within two months of diagnosis

Since spontaneous closure was observed in eyes with small iFTMHs (*n* = 124), we plotted this data on a Kaplan–Meier curve and found that approximately 25% of the iFTMHs closed spontaneously within two months. For a diameter of < 150 µm (*n* = 48), the frequency of the spontaneous closure increased to 55% of eyes within that time period (Fig. 2).

## Discussion

The study included 338 eyes with iFTMHs and revealed a spontaneous closure rate of 9.2% at a median of 44 days after the diagnosis. All eyes with spontaneous improvement had small iFTMHs.

Previous retrospective studies using OCT imaging for iFTMH diagnosis reported spontaneous closure in 2,7–3,5% of eyes, while the mean time to spontaneous closure were 2, 2.5, and 5 months [17–19]. The time to closure was similar in our large cohort, however, we found a higher rate of spontaneous closure (9.2%). This discrepancy may be explained by the different study populations. In our study, eyes with spontaneous closure had an average hole diameter of 98.9 µm, which was smaller compared to 201 µm, 195 µm and 162 µm found in previous studies. This might suggest that patients with smaller iFTMH are underrepresented in the other studies, leading to a lower spontaneous closure rate.

One of the prospective studies investigating the effect of ocriplasmin in eyes with vitreomacular traction or iFTMH found in the control group a spontaneous closure rate of 15.4% (4/26 eyes with iFTMH) at month three without re-opening in the 24-month follow-up period [8, 23, 24].

In our study population, spontaneous closure was found only in eyes with small iFTMHs, whereas in the mentioned retrospective studies, few eyes had diameters greater than 250 µm [17–19]. Smaller iFTMHs have been reported to have a higher likelihood of spontaneous closure. Our study found a significant correlation between hole diameter and spontaneous closure.

There are also reports regarding the spontaneous re-opening between three months and three years after achieving complete iFTMH closure with or without surgical intervention [10, 25–27]. In our study population, we found re-opening of spontaneously closed iFTMH in five patients with a median of 4 months after closure. Considering the relatively short time span until reopening, the continuation of the regular follow-up examinations is important. In our clinic, patients were advised to have follow-up examinations at 2-week intervals for 6 weeks after the initial diagnosis. Subsequently, ophthalmological examinations were performed alternately with local ophthalmologists at four-week intervals, provided an OCT examination was available. As all patients in our study had full health insurance, it is unlikely that any missed follow-up examinations were due to monetary reasons. Although we cannot be certain, we assume that patients were referred if any morphological or functional worsening was noticed by the local ophthalmologists. All patients with a re-opening after spontaneous closure underwent macular surgery and showed closure of the iFTMH. Due to the small number of observed re-opened iFTMHs, further statistical analysis of OCT features was not feasible. In the aforementioned retrospective studies and the prospective OASIS study, only one re-opening was described which might be due to the lower number of observed spontaneous closed iFTMHs compared to our study [17–19, 23, 25, 28].

The exact mechanism of spontaneous closure of iFTMH remains unknown. One explanation is that the release of vitreous traction leads to decreased vertical forces and to a flattening and convergence of the hole edges [29]. However, since the proportion of eyes with and without vitreofoveal traction was comparable in both groups (30% in the no spontaneous closure group, and 45% in the spontaneous closure group, *p* = 0.103), other mechanisms should also be considered. Furthermore, closure of the iFTMH with persistent vitreofoveal traction is also reported [18]. Consequently, we would consider the release of vitreofoveal traction favorable for spontaneous closure, although this cannot be generalized for all cases with vitreofoveal traction.

Interestingly, in case reports of secondary full-thickness macular holes associated with cystoid macular edema in uveitis, ocular trauma, post-laser inflammation, or Irvine-Gass syndrome, the resolution of the intraretinal pseudocysts is thought to have contributed to spontaneous closure [10, 26, 30–36].

**Fig. 3:**
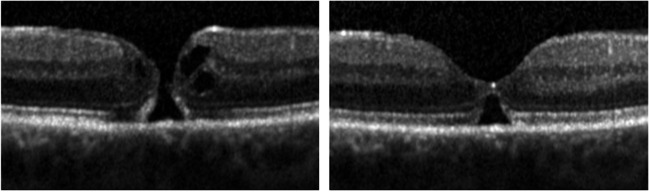
Spontaneous closure of an idiopathic full-thickness macular hole with bridging phenomenon in a 57-year-old lady 52 days after diagnosis

The formation of a bridge-like structure at the edges of the hole is thought to be a reparative reaction of the Müller cells preceding the spontaneous closure [37–39]. In line with these reports, many eyes with spontaneous closure in our study presented with a bridging phenomenon in the OCT (Fig. 3).

So far, limited information is known about the time course of spontaneous closure of the small iFTMHs. Based on the data presented, approximately 25% of small iFTMHs (diameter ≤ 250 µm) and around 50% of iFTMHs with diameters < 150 µm can close spontaneously within two months of diagnosis. Therefore, in patients with small iFTMHs and relatively good vision a „watchful waiting" approach with short follow-up regime may be offered to some patients after careful considerations of all circumstances. However, a recent meta-analysis estimated a minor vision loss of 0.008 logMAR per month of additional waiting for surgery [20]. As this study included iFTMHs of all sizes, and small iFTMHs have a higher surgical success rate, extrapolating the degree of vision loss for small iFTMHs only is difficult [20, 21].

One of the main limitations of this study is its retrospective study design. However, we believe that our analysis is rather underestimating spontaneous closure rates because patients with spontaneous BCVA improvement may not seek ophthalmological examination in the first place or are more likely to miss follow-up appointments. Moreover, the time of the iFTMH closure is likely overestimated, as spontaneous closure was diagnosed on the day of the OCT examination and not at the time of subjective BCVA improvement. However, it is notable that the time of diagnosis based on the OCT examination is not the time at which the iFTMH actually develops. An alternative to our approach is using the onset of symptoms as the time of diagnosis. Nevertheless, we have decided against this, as not all patients are able to state the onset of symptoms precisely enough (e.g. if the healthy partner eye is dominant). Therefore, caution should be applied when generalizing these results.

In summary, this study revealed spontaneous closure of small iFTMHs in a relatively high percentage of patients within two months of diagnosis in our cohort. Considering that the established standard therapy for iFTHMs is a surgical approach, this information could be relevant to clinical practice and future studies. While our data cannot be used to predict the course of an individual patient's disease with high accuracy, future investigations are required to validate the conclusions drawn from this study and to help establish validated treatment recommendations for patients with small iFTMHs in the clinical routine.
